# Protective factors of prenatal depression, anxiety and stress: a network analysis of Bernstein’s strengths model

**DOI:** 10.3389/fpsyt.2025.1572142

**Published:** 2025-09-29

**Authors:** Sarolta Vasvári, Mónika Miklósi

**Affiliations:** ^1^ Doctoral School of Psychology, Eötvös Loránd University (ELTE), Budapest, Hungary; ^2^ Institute of Psychology, Eötvös Loránd University, Budapest, Hungary; ^3^ Centre of Mental Health, Heim Pál National Pediatric Institute, Budapest, Hungary

**Keywords:** prenatal, depression, anxiety, stress, personal strengths, social support, relationship satisfaction, protective factors

## Abstract

**Objective:**

Symptoms of depression, anxiety, and stress are highly prevalent during pregnancy and have long-lasting negative effects on the mother and the child. Identifying the complex interrelations of protective factors in a theory-driven way is crucial for designing effective psychosocial interventions. The present study aims to explore the effect of personal strengths, and social and environmental factors on depression, anxiety and stress in a sample of pregnant women using a network model.

**Method:**

A sample of 346 pregnant women (gestation age range: 12–40 weeks) completed an online questionnaire, which included the Depression Anxiety Stress Scale (DASS), the World Health Organization Quality of Life Assessment, 26-item version (WHOQOL-BREF), the Multidimensional Scale of Perceived Social Support (MSPSS), the Relationship Assessment Scale (RAS), and the Bernstein’s Strengths Scale (BSS).

**Results:**

The nodes representing DASS subscales were highly and positively interconnected. Depression demonstrated negative associations with relationship satisfaction, social support, physical and environmental health, emotional balance and self-confidence. Anxiety had negative connections with physical and environmental health and resilience; however, it showed positive relationships with imagination and self-assertion. Stress was significantly and negatively related to physical health, emotional balance, resilience and gratitude. Depression, wisdom, and identity had the highest strength centrality, followed by emotional balance and resilience, indicating that these are the most influential nodes in the network.

**Conclusion:**

The findings of this study indicate that multifactorial interventions targeting social, physical, environmental factors, and personal strengths, particularly resilience, emotional balance, and self-confidence, hold potential as effective strategies to enhance maternal mental health during pregnancy.

## 1 Introduction

Approximately one-fifth of pregnant women experience symptoms of depression or anxiety, which can adversely affect the health of both the mother and the child (1–5). Symptoms of anxiety have been reported by 18.2% of women in the first trimester, 19.1% in the second trimester, and the prevalence increased up to 24.6% in the third trimester (1). Notable differences were found across European countries, ranging from 7.7% in Poland to 36.5% in Italy (6). The global prevalence of antenatal depression was found to be 20.7% and this prevalence did not significantly differ across pregnancy trimesters (2). Caffieri et al.’s (7) umbrella review covering the period of the pandemic reports clear regional differences: prenatal depression was highest in Africa (44%) and the lowest in South America (25%), while antenatal anxiety peaked in Oceania (42%) and Europe (41%), and the lowest in Asia (23%). The authors observe that the heterogeneity is partly driven by country of residence and by differences in assessment tools and cut-offs, underscoring how regional and cultural context and measurement practices shape observed prevalence. A recent study on a large, Hungarian sample using self-report, revealed that 12.5% of pregnant women in their third trimester were likely experiencing depression, and 15.8% showed signs of an anxiety disorder (3). Moreover, the co-occurrence of anxiety and mood disorders is notably high, and elevated anxiety levels during pregnancy serve as the most robust predictor of depression (8, 9). Prenatal depression is a significant predictor of postpartum depression, which may occur in up to 22% of women in the year following childbirth (10). Anxiety and depression during pregnancy can increase the risk of preterm birth and lower developmental levels in the infant (11, 12). Maternal prenatal stress, anxiety and depression may adversely influence the cognitive, behavioral, psychomotor (13), and socio-emotional development of the child (14). Parental stress has been shown to result in a multitude of adverse outcomes in the offspring, including cognitive deficits, emotional dysregulation, and an increased risk for psychopathology (15).

A substantial body of research has been dedicated to examining the various risk and protective factors associated with antenatal anxiety and depression (16). The extant literature suggests that socio-demographic factors, including younger age, less education, non-marital status, financial difficulties (3, 8, 17, 18), and non-employment (19, 20), may increase the risk of anxiety and depression during pregnancy. Past adverse life events, such as a history of abuse or domestic violence (21), past pregnancy complications or loss, as well as actual life adversities, high perceived stress and low satisfaction with life (8, 19) are also well-known risk factors for anxiety and depression during pregnancy. Somatic and psychiatric risk factors include personal (8) or family history of mental illness (22), alcohol use (23), smoking (17, 24), somatic disease (25), sleep disorder (22), and inactive lifestyle during pregnancy (26). Moreover, a number of significant pregnancy-related factors have been identified, including unwanted/unplanned or complicated pregnancy (8, 19, 27), multiparity, and hyperemesis gravidarum (22). Physiological factors, i.e. cortisol, amylase, pro-inflammatory cytokines, intrauterine artery resistance (19), or thyroid function (28) have also been identified as potential risk factors.

The most consistent findings pertain to the protective factors associated with social determinants, including the presence of a partner, social support (8, 19, 24, 29), and relationship satisfaction (25, 30, 31). Conversely, marital conflict has been demonstrated to function as a risk factor (17). The significance of social support and relationship quality in maintaining mental well-being during pregnancy has also been demonstrated in cross-cultural contexts (18, 32–35). A paucity of research exists on psychological risk and protective factors. Research has identified associations between psychoticism, neuroticism (36), external locus of control (19, 37), and negative cognitive style (8) with elevated levels of anxiety and depression symptoms during pregnancy. Conversely, active coping emerged as a protective factor (36). In one study, more individualistic women were also found to be less likely to show prenatal depressive symptoms (38).

A body of research has centered on personal strengths, defined as behavioral, cognitive, or emotional capacities that facilitate optimal functioning and performance in achieving valued outcomes (39). Results suggest that self-confidence (40), self-efficacy (24, 41), sense of mastery (42), resilience (32, 41, 43), and hope (44) have been associated with better mental health during pregnancy. However, there is a paucity of theory-based, comprehensive research on personal strengths as protective factors of depression, anxiety, and stress during pregnancy. Such research would foster the development of positive psychological interventions aiming at improving the mental health of pregnant women (45).

Advancing Young’s schema theory (46), David Bernstein (47) recently proposed a concept of personal strengths and developed a positive psychological schema therapy intervention to support the healthy adult self. The healthy adult self is defined as the capacity of an individual to use adaptive mechanisms, thereby fostering autonomy and responsibility, and cultivating a positive relationship with oneself and one’s environment (48). Bernstein (47) has delineated 16 strengths of the healthy adult self, which are subsequently categorized into four overarching higher-order factors. Self-directedness comprises strengths that empower individuals to determine their life path, including identity, self-reflection, self-confidence, self-assertion, and imagination/creativity. The self-regulation factor encompasses strengths such as emotional balance, resilience, self-control, self-care, and reality testing, collectively enabling effective regulation of emotions, thoughts, and behavior. The connection factor encompasses strengths such as empathy, compassion, humour, and responsibility, that serve to establish meaningful reciprocal relationships. Finally, the transcendence factor refers to higher-order human aspirations or the pursuit of meaning in life and social relationships. Two such strengths are gratitude and wisdom (**Table 1**).

**Table 1:** Bernstein’s Strengths Model.

Factors/Strengths	Sample item of BSS	Description
Self-directedness factor		
Identity	I know who I am.	The individual possesses a clear sense of self, reflected in the qualities that define their unique identity.
Self-reflection	I take time to self-reflect.	The individual is capable of self-reflection, regularly examining their thoughts, feelings, and behavior.
Self-confidence	I trust my judgement.	The individual demonstrates self-confidence and trust in their abilities, judgment, and personal qualities.
Self-assertion	I say what I need or want.	The individual can assert their rights, needs, and beliefs respectfully.
Imagination	I come up with creative solutions.	The person shows creativity and effective problem-solving, using cognitive skills to explore ideas and envision future possibilities.
Self-regulation factor		
Emotional balance	I keep my emotions in balance.	The person maintains emotional balance and responds to situations in a calm and steady way.
Self-control^#^	I stop and think before acting.	The individual is able to pause and think before acting, showing the capacity to delay immediate desires and manage frustration effectively.
Self-care	I have healthy habits.	The individual actively cares for their emotional and physical well-being.
Reality testing	I am in touch with reality.	The person evaluates the validity of their thoughts, emotions, and perceptions through a rational and thoughtful lens.
Resilience	I can handle stress.	The person handles challenges well by staying calm and adjusting to the situation.
Connection factor		
Empathy	I feel what others are feeling.	The individual demonstrates empathy by recognizing and resonating with the feelings of others.
Compassion	I show kindness to others.	The individual expresses kindness and a genuine willingness to support others.
Humour	I have a sense of humour.	The person brings a sense of playfulness and humour, often contributing to a positive atmosphere.
Responsibility	I am responsible.	The individual demonstrates a consistent pattern of reliability and responsibility.
Transcendence factor		
Gratitude	I am thankful for what I receive.	The person expresses gratitude and appreciation for what they have, rather than taking things for granted.
Wisdom	I seek wisdom to live by.	The person is looking for truth and understanding, makes wise decisions, and grows through life experiences.

BSS: Bernstein’s Strengths Scale.

^#^ The Self-control factor was left out of our analysis because of reliability issues.

This model is closely linked to the core functional domains of the Diagnostic and Statistical Manual of Mental Disorders, Fifth Edition (DSM-5 (49)) Alternative Model for Personality Disorders (AMPD), which evaluates self-functioning (identity, self-direction) and interpersonal functioning (empathy, intimacy) on a continuum from healthy to severely impaired (Criterion A). It also corresponds with the concept of healthy personality functioning in the International Classification of Diseases 11th Revision (ICD-11) (50). In line with those models, the “healthy adult mode” is viewed as a transdiagnostic core construct which integrates key self- and interpersonal functions that are essential for adaptive functioning across a wide range of mental health conditions (51).

Unlike widely used positive psychology models such as Seligman’s PERMA framework (52), the VIA classification (53), or Self-Determination Theory (54), the Bernstein model was developed with strong clinical relevance. While positive psychology focuses on well-being, flourishing, and character strengths in the general population, Bernstein’s model takes a more clinical perspective, tailoring for therapeutic settings and vulnerable populations, and highlighting how personal strengths function as protective and restorative resources. Bernstein not only developed a theoretical framework of personal strengths but also designed a comprehensive set of therapeutic tools aimed at strengthening these capacities in clinical practice (47). The iMood System (47) offers a structured yet flexible way to support clients in reconnecting with their healthy adult self. Bernstein’s strengths-focused approach has been gaining increasing popularity among therapists worldwide.

Despite its growing use in psychotherapy, empirical research on the Bernstein Strengths-based approach remains limited, however. In our previous research on a general, non-clinical adult sample (55) on the validity of the strengths model, we found that mental health was related to several interconnected strengths, with the strongest connection being demonstrated to gratitude. Furthermore, wisdom and self-confidence emerged as the most influential nodes in a network model.

This initial research supported Bernstein’s model as a promising approach to exploring personal strengths in a comprehensive way. However, given its growing popularity and rapid integration into clinical practice, and the limited availability of empirical evidence, further research is warranted in different settings and populations. Therefore, as a second step toward the exploration of the usefulness of the model, this study aimed to assess the associations between personal strengths, social and environmental factors, and depression, anxiety, and stress in a sample of pregnant women. In the contemporary era, the lives of women expecting childbirth and preparing for the parental role are significantly affected by technological advances (56) and social changes (57). Consequently, focusing on internal resources can be particularly important. We applied a network analysis, which provides a dynamic, interconnected, and holistic perspective on the protective factors associated with depression, anxiety, and stress. This approach is particularly valuable in capturing complex interactions between various factors and identifying indirect relationships. More precisely, we aimed to identify specific protective factors for depression, anxiety and stress, taking into account their interconnected nature.

It was hypothesized that social factors and personal strengths would be associated with mental health. In accordance with prior research, it was anticipated that there would be negative correlations between social support (8, 19, 24, 29), relationship satisfaction (25, 30, 31), self-confidence (40), resilience (32, 41, 43), gratitude (55), and psychopathological symptoms. It was also hypothesized that wisdom and self-confidence would be the most influential nodes in the network (55).

## 2 Method

### 2.1 Sample and procedures

The study was conducted as part of a longitudinal research project investigating pregnant women’s psychological well-being, personal resources, social support, and relationship satisfaction, as well as how these factors influence their later satisfaction with their parental role and their relationship with their newborn child. Ethical approval was obtained by the Institutional Research Ethics Committee of the Psychological Institute, Eötvös Loránd University (Nr. 2023/53-2). Data collection was conducted online through a questionnaire survey using the Qualtrics platform. Participants were recruited through prenatal social media groups and maternal health networks. Participants were recruited from the 12th week of pregnancy onward to avoid the ethically sensitive early gestational period, during which miscarriage risk is elevated (58). This timing also aligns with clinical protocols (59, 60), ensuring a more stable psychological baseline and reducing dropout due to early pregnancy-related symptoms (60). Participants indicated that they were not under neurological or psychiatric treatment at the time of the study. They were enrolled in the study following informed consent. The information and consent form provided detailed information about the precise objectives of the study, the voluntary nature of participation, the anonymity of data collection, and the confidential handling and use of the information gathered during the research.

Three hundred and seventy-eight participants filled out the questionnaire, and 32 were excluded due to incomplete data (missing data > 10%). Thus, the data of 346 pregnant women were analyzed. The mean age was 32.23 years (*SD*=4.64, range: 21-46 years). One hundred and twenty-six (36.4%) of the participants lived in the capital, 145 (41.9%) of them lived in urban areas and 75 (21.7%) in rural areas of the country. The majority of the women were married (*N*=308, 89.0%) or lived in a relationship (*N*=33, 9.5%). Three-fourths of them (*N*=258, 74.5%) had a university or college degree, 75 (21.7%) had a medium level of education (12 years), and 13 (3.8%) had lower levels of education. About half of the participants (*N*=178, 51.4%) were economically active. One hundred and twenty-seven (36.7%) reported a good financial state, while 179 (51.7%) had satisfactory financial conditions, and 40 (11.6%) reported having financial problems. Almost two-thirds of the participants (*N*=228, 65.9%) were first-time expectant women. According to the established inclusion criteria, all participants had a gestational age exceeding 12 weeks. However, specific details regarding gestational age were available for a subset of 158 participants who consented to undergo follow-up assessments (*M*=27.28 weeks, *SD*=8.13, *range:* 12–40 weeks). Fifty-four (34%) participants were in the second trimester, while 104 (66%) of them were in the third trimester.

### 2.2 Measure

The Depression Anxiety Stress Scale, 21-item version (DASS (61, 62);) is a short self-report tool designed to assess negative emotional states quantitatively. Comprising three subscales of 7 items each, it evaluates depression (DASS-D), anxiety (DASS-A), and stress (DASS-S) levels. Participants respond on a 4-point Likert scale ranging from 0 (“does not apply to me at all”) to 3 (“applies to me very much”), where higher scores indicate more severe symptoms. Cut-off scores suggested by the original authors were used for reporting the prevalence of elevated levels of depression (DASS-D scores > 9), anxiety (DASS-A scores > 7), and stress (DASS-S scores > 14). Good psychometric properties have been reported for the original version (62). The DASS-21 has also been validated in pregnant women, showing adequate internal consistency and stability (63). In our data, internal consistencies of the subscales were good to very good (**Table 2**).

**Table 2:** Descriptive statistics and reliabilities of study variables, as well as bivariate relationships of DASS subscales with protective factors.

Scale	α	Mean	SD	Range	Skew	Person’s r		
						DASS depression	DASS anxiety	DASS stress
DASS Depression	0.856	3.74	3.68	0 — 19	1.491	—		
DASS Anxiety	0.700	4.06	3.26	0 — 16	1.006	0.588*	—	
DASS Stress	0.850	6.71	4.28	0 — 21	0.808	0.693*	0.567*	—
WHOQOL-BREF Physical	0.819	25.99	4.65	11 — 35	-0.503	-0.467*	-0.420*	-0.418*
WHOQOL-BREF Environmental	0.776	31.66	4.55	15 — 40	-0.500	-0.394*	-0.282*	-0.300*
RAS	0.843	35.29	4.73	16 — 40	-1.830	-0.379*	-0.207*	-0.318*
MSPSS	0.887	43.42	6.41	21 — 50	-1.159	-0.412*	-0.182*	-0.294*
BSS Identity	0.741	12.02	2.14	6 — 15	-0.540	-0.331*	-0.166	-0.288*
BSS Self-reflection	0.766	11.25	2.52	3 — 15	-0.510	-0.186*	-0.100	-0.170
BSS Self-confidence	0.839	11.30	2.47	4 — 15	-0.448	-0.423*	-0.258*	-0.354*
BSS Self-assertion	0.833	11.33	2.73	4 — 15	-0.462	-0.203*	-0.054	-0.147
BSS Imagination	0.640	11.60	2.13	3 — 15	-0.555	-0.226*	-0.067	-0.178*
BSS Emotional balance	0.796	9.80	2.49	3 — 15	-0.163	-0.505*	-0.319*	-0.502*
BSS Self-care	0.605	10.82	2.16	5 — 15	-0.176	-0.276*	-0.167*	-0.313*
BSS Reality testing	0.623	11.75	2.00	5 — 15	-0.448	-0.329*	-0.176*	-0.273*
BSS Empathy	0.878	12.97	2.25	6 — 15	-1.034	-0.068	-0.032	0.022
BSS Compassion	0.664	8.66	1.37	4 — 10	-0.945	-0.130	-0.070	-0.041
BSS Resilience	0.606	10.94	2.02	3 — 15	-0.466	-0.418*	-0.315*	-0.391*
BSS Humour	0.693	12.85	2.01	5 — 15	-1.005	-0.251*	-0.109	-0.136
BSS Responsibility	0.681	13.40	1.66	7 — 15	-1.043	-0.196*	-0.101	-0.143
BSS Gratitude	0.861	13.61	1.91	6 — 15	-1.560	-0.260*	-0.132	-0.250*
BSS Wisdom	0.618	12.10	1.99	6 — 15	-0.487	-0.306*	-0.157	-0.240*

*N*=346. DASS: Depression Anxiety Stress Scale. WHOQOL-BREF: The World Health Organization Quality of Life Assessment, 26-item version. RAS: Relationship Assessment Scale. MSPSS: Multidimensional Scale of Perceived Social Support. BSS: Bernstein’s Strengths Scale. *SD*: standard deviation. * *p*<0.002 (α’=0.05/22).

The World Health Organization Quality of Life Assessment, 26-item version (WHOQOL-BREF (64),) is a concise, self-administered adaptation of the WHOQOL-100 designed to assess the quality of life universally across cultures. This 26-item questionnaire measures subjective quality of life in several domains. We used the physical health domains, which encompass seven items addressing work capacity, daily activities, stamina, mobility, pain, sleep, and medical needs, and the environmental quality domain, which is gauged through eight items assessing safety, financial resources, access to information, leisure activities, living conditions, healthcare access, and transport. Responses utilize a 5-point Likert scale; higher scores represent better quality of life. The scale has been validated in the pregnant population as well (65). The Hungarian version, adapted by Paulik and colleagues (66), showed good internal consistency (Cronbach’s alpha > 0.7). In our sample, internal reliabilities were good to very good (**Table 2**).

The Multidimensional Scale of Perceived Social Support (MSPSS), developed by Zimet et al. (67), is a brief self-report questionnaire aimed at assessing perceived social support. It includes 12 items, each rated on a 7-point Likert scale from ‘very strongly disagree’ (1) to ‘very strongly agree’ (7). The MSPSS comprises three subscales (Family, Friends, and Significant Others) with four items each. Scores are obtained by summing the items on each subscale, with higher scores indicating greater perceived social support. The MSPSS has also been validated in pregnant women (68). Good psychometric properties of the Hungarian version have been reported (69). In our data, Cronbach’s alpha was 0.887.

The Relationship Assessment Scale (RAS (70),) originally consisted of 7 questions, with the 8th supplementary question being added in the Hungarian version (71). Respondents provide answers on a five-point Likert scale. Higher scores on the scale indicate greater relationship satisfaction. Both the original and the Hungarian version had internal consistencies. In our sample, Cronbach’s alpha was 0.843.

The Bernstein’s Strengths Scale (BSS (47),) assesses 16 personal strengths corresponding to Bernstein’s Strengths Model. The BSS consists of 48 Likert-type items; respondents are asked to judge what strengths they can rely on in difficult situations by indicating on a 5-point Likert scale how much they agree with each statement (from 1=not at all agree, to 5=fully agree). Up to now, the BSS has been translated into more than 25 languages and has become widely used in clinical settings. However, to our knowledge, our research group was the first to evaluate its psychometric properties (55). The Hungarian version was created using a back-translational process, and the factorial validity of the scale was explored in a non-clinical adult sample. Although the data demonstrated an acceptable fit to the 16-factor model, the self-control subscale was excluded due to reliability concerns. The final model, incorporating 15 factors, demonstrated optimal fit (χ^2^(797) = 1130.149, *p* < .001, RMSEA = 0.028, 90% CI [0.024, 0.032], SRMR = 0.049, CFI = 0.993) (55). Similarly, in the current data, 15 of the 16 subscales showed acceptable to good internal consistencies (**Table 2**). Cronbach’s alpha of the self-control subscale fell below the acceptable cut-off (0.6); therefore, we left it out of further analyses.

### 2.3 Statistical analyses

Missing values<10% were imputed by using a regression-based method. Descriptive statistics and internal consistencies of the scales are reported. The associations between DASS subscales and protective factors were assessed by means of Pearson’s correlational coefficient. A series of independent t-tests and ANOVAs was conducted to analyze the effects of demographic variables on symptoms of depression, anxiety, and stress, using Bonferroni correction for multiple statistical tests.

We used a Gaussian graphical model with a graphical lasso method based on an extended Bayesian inference criterion estimation approach (72) to conduct a network analysis, using JASP statistical software (73). Each of the 22 nodes corresponded to the subscale scores of DASS, WHOQOL-BREF, and BSS, as well as the total scores of the RAS and MSPSS. The edges denote the partial correlations between the scores of the factors. Centrality indices (74) were used to identify the most influential nodes in the network.

## 3 Results

### 3.1 Preliminary analyses

The descriptive statistics and reliabilities of the scales are presented in **Table 2**. In the sample, 9.0% (*N*=31), 15.6% (*N*=54), and 6.1% (*N*=21) of the participants reported elevated levels of depression (DASS-D scores > 9), anxiety (DASS-A scores > 7), and stress (DASS-S scores > 14), respectively. Altogether, 72 (20.8%) women reported elevated levels of distress in at least one domain. In the subsample with information about gestation age, no differences were found between women in the second and third trimesters.

We assessed the impact of demographics on study variables, using Bonferroni correction for multiple tests (α’=0.005/19 = 0.003). Age was negatively related to gratitude (*r*=-0.198, *p*<0.001). No differences were found across residence locations. Participants with high levels of education reported higher environmental health than participants with low/medium levels of education (*M_high_* =32.11, *SD_high_* =4.30, *M_medium/low_* =30.34, *SD_medium/low_* =5.00, *t*(344)=3.189, *p*=0.002, *d*=0.394). Economically active women reported higher levels of physical health (*M_active_* =26.87, *SD_active_* =4.35, *M_passive_* =25.06, *SD_passive_* =4.79, *t*(344)=3.684, *p*<0.001, *d*=0.396), environmental health (*M_active_* =32.54, *SD_active_* =4.08, *M_passive_* =30.73, *SD_passive_* =4.84, *t*(344)=3.775, *p*<0.001, *d*=0.406), emotional balance (*M_active_* =10.23, *SD_active_* =2.40, *M_passive_* =9.35, *SD_passive_* =2.51, *t*(344)=3.353, *p*<0.001, *d*=0.361), self-care (*M_active_* =11.20, *SD_active_* =2.08, *M_passive_* =10.42, *SD_passive_* =2.18, *t*(344)=3.380, *p*<0.001, *d*=0.364), and resilience (*M_active_* =11.36, *SD_active_* =1.88, *M_passive_* =10.49, *SD_passive_* =2.07, *t*(344)=4.081, *p*<0.001, *d*=0.439), and lower levels of stress (*M_active_* =5.91, *SD_active_* =3.82, *M_passive_* =7.55, *SD_passive_* =4.59, *t*(344)=-3.615, *p*<0.001, *d*=0.389) than women who were not economically active. First-time expectant women reported higher levels of self-care than second- or more expectant women (*M_first_* =11.09, *SD_first_* =2.06, *M_more_* =10.30, *SD_more_* =2.26, *t*(344)=3.292, *p*=0.001, *d*=0.373). The gestation age showed a significant, albeit small, negative correlation with WHOQOL-BREF Physical Health (*r*=-0.210, *p*=0.008). No differences were found between women in the second and third trimesters.

### 3.2 Results of the network analysis

**Figure 1:**
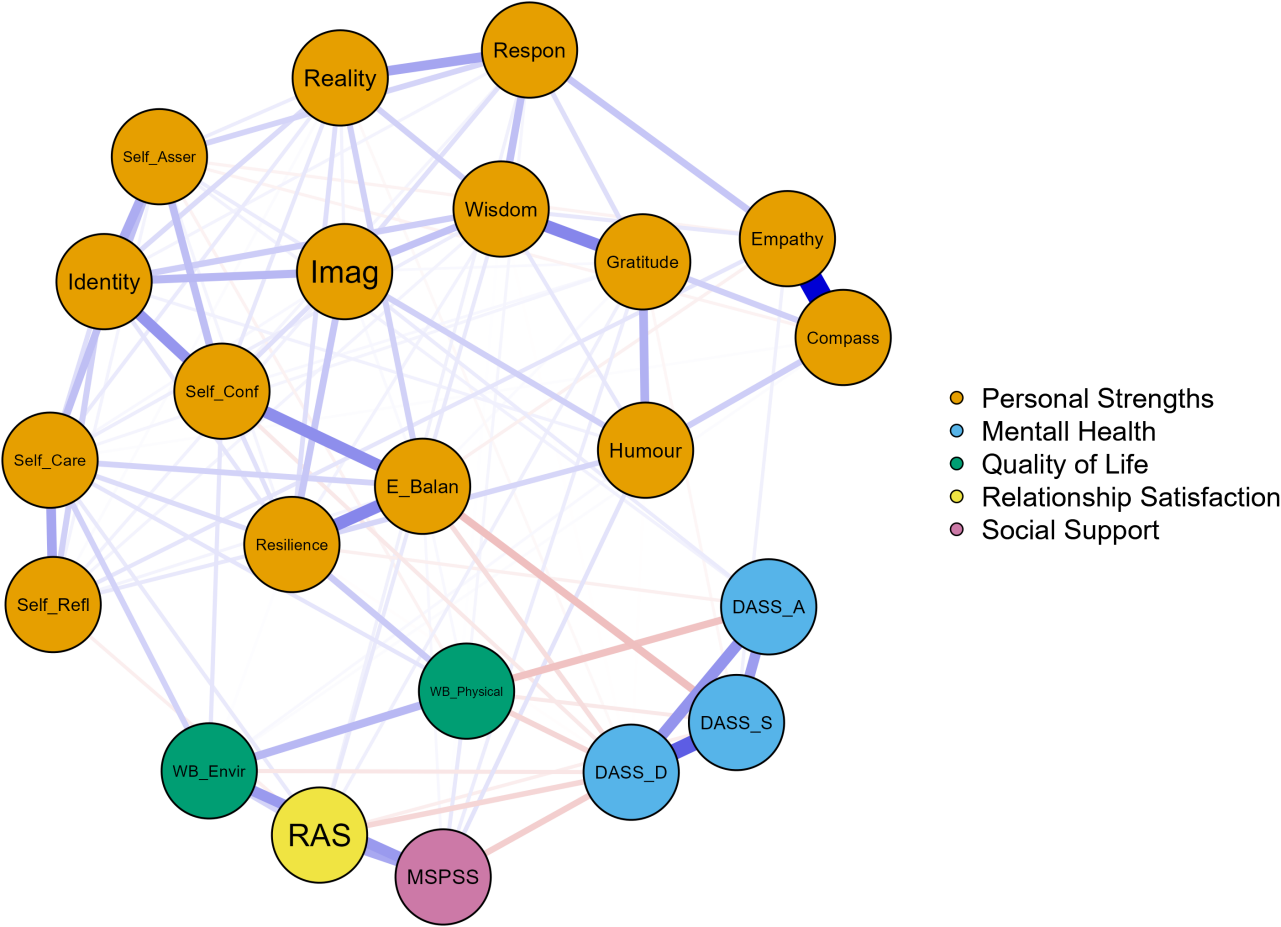
The network model. *N*=346. Sparsity=0.498 (= 1 – 2*e/[n*(*n*-1)], where *e* denotes the number of edges and *n* represents the number of nodes.) Blue edges represent positive relations, and red edges represent negative relations. Thicker edges indicate stronger connections. DASS_D, Depression Anxiety Stress Scale, depression subscale. DASS_A, Depression Anxiety Stress Scale, anxiety subscale. DASS_S, Depression Anxiety Stress Scale, stress subscale. WB_Physical, The World Health Organization Quality of Life Assessment, 26-item version (WHOQOL-BREF), Physical well-being. WB_Environmental, The World Health Organization Quality of Life Assessment, 26-item version (WHOQOL-BREF), Environmental well-being. RAS, Relationship Assessment Scale. MSPSS, Multidimensional Scale of Perceived Social Support. BSS, Bernstein’s Strengths Scale. Self-Refl, Self-reflection, Self-Asser, Self-assertion. Self-Conf, Self-confidence. E-balan, Emotional balance. Imag, Imagination/creativity. Reality, Reality testing. Compass, Compassion. Respon, Responsibility.

The sparsity of the network (**Figure 1**) was 0.498. Out of a total of 231 possible edges, 116 edges were observed to have nonzero values. This suggests that the network exhibited a moderate level of connectivity, with a considerable number of existing connections between nodes. The edge-weight correlation plot from the case-drop bootstrap indicated that the edge weights were robust and that the overall network structure remained stable under subsampling.

The strongest edge connected empathy and compassion, followed by the edges between depression and stress, wisdom and gratitude, emotional balance and resilience, self-confidence, and identity.

The nodes representing DASS subscales were highly and positively interconnected, indicating that symptoms of depression, anxiety and stress are often comorbid.

Depression was negatively related to social protective factors. It was also negatively associated with physical and environmental health. Among personal strengths, depression had significant negative edges with emotional balance, self-confidence, reality testing, resilience, humour, and wisdom.

Anxiety had the strongest negative edge with physical health. It was also significantly and negatively connected with environmental health, as well as resilience; however, it showed positive relationships with imagination and self-assertion.

Finally, stress was significantly and negatively related to physical health, relationship satisfaction, as well as emotional balance, resilience and gratitude. However, a weak but significant positive association was found between stress and empathy.

Expected influence centrality measures (74) were checked to estimate the potential impact of the influences from the subscale scores within the network. Wisdom and identity demonstrated the highest positive expected influence values in the network, indicating that these nodes activate positively connected nodes and suppress negatively connected ones, potentially spreading positive influence. On the other hand, depression and physical health had the highest negative expective influence values, suggesting their central role in symptom maintenance or intensification (**Table 3**). Strength centrality measures (75) were checked to determine the overall influence of each item within the network. Depression, wisdom and identity had the highest strength centrality, followed by emotional balance and resilience, indicating that these are the most influential nodes in the network.

**Table 3:** Significant edge weights with DASS nodes and (z-scored) centrality indices.

Nodes of the network	*Significant edge weights*			*Betweenness^#^*	*Closeness^#^*	*Strength*	*Expected Influence*
	*DASS Depression*	*DASS Anxiety*	*DASS Stress*				
DASS Depression	—	0.261	0.395	-0.315	-0.404	1.577	-1.952
DASS Anxiety	0.261	—	0.239	-1.060	-1.016	-0.890	-1.046
DASS Stress	0.395	0.239	—	1.473	0.266	0.116	-1.031
WHOQOL-BREF Physical	-0.103	-0.148	-0.055	-0.687	-0.202	-0.751	-1.905
WHOQOL-BREF Environmental	-0.061	-0.007		-0.091	-0.616	-0.782	-0.175
RAS	-0.099		-0.043	-0.017	-0.673	-0.914	-0.589
MSPSS	-0.120			-0.985	-1.546	-1.405	-1.275
BSS Identity				0.430	1.097	1.354	1.462
BSS Self-reflection				-0.985	-0.547	-1.752	-0.553
BSS Self-confidence	-0.057			0.281	1.299	0.777	0.767
BSS Self-assertion		0.033		-0.166	0.681	-0.154	0.002
BSS Imagination		0.048		-0.762	0.654	0.093	0.752
BSS Emotional balance	-0.083		-0.157	3.260	2.195	1.360	-0.317
BSS Self-care				0.058	0.244	0.540	1.003
BSS Reality testing	-0.017			-0.389	-0.006	-0.607	0.250
BSS Empathy			0.037	-0.762	-1.480	0.335	0.443
BSS Compassion				-0.538	-1.447	0.294	0.666
BSS Resilience	-0.004	-0.038	-0.001	1.249	1.721	1.156	1.076
BSS Humour	-0.003			0.728	0.097	-1.297	-0.050
BSS Responsibility				-0.240	-0.180	-0.193	0.590
BSS Gratitude			-0.022	-0.687	-0.397	-0.368	0.348
BSS Wisdom	-0.002			0.207	0.258	1.512	1.536

*N*=346. DASS, Depression Anxiety Stress Scale. WHOQOL-BREF, The World Health Organization Quality of Life Assessment, 26-item version. RAS, Relationship Assessment Scale. MSPSS, Multidimensional Scale of Perceived Social Support. BSS, Bernstein’s Strengths Scale. *^#^* Betweenness and Closeness centrality indices did not demonstrate sufficient stability (< 0.25); consequently, we did not interpret them.

Emotional balance and resilience had a strong positive correlation, and both strengths were highly interrelated with several other strengths. Emotional balance was positively connected to self-confidence, reality-testing, self-care, gratitude, imagination and wisdom. At the same time, resilience demonstrated positive associations with identity, self-reflection, self-confidence, self-assertion, imagination, self-care, humour, and wisdom.

## 4 Discussion

Pregnancy is associated with a high prevalence of depression, anxiety, and stress. Specifically, 9% of the sample reported elevated levels of depression, which is slightly lower than the prevalence rates found in a recent Hungarian study (3). However, the prevalence of elevated anxiety levels was equivalent (15%). These symptoms have been demonstrated to have long-lasting detrimental effects on maternal and offspring health outcomes (8, 9, 11–15). The identification of risk and protective factors is crucial for the promotion of mental health in pregnant women. In a comprehensive, theory-based model, we assessed the interconnection of environmental, social and personal protective factors of depression, anxiety, and stress in a sample of pregnant women. We developed a network model, which helps identify not just individual predictors but also how they are dynamically interconnected within the system, providing a more nuanced understanding of the causes of psychopathology. The findings of the network analysis can inform multifactorial interventions by identifying the most influential nodes and targeting them collectively.

### 4.1 Possible intervention targets

In line with previous research (8, 9), results revealed that distress factors are highly interrelated, with depression demonstrating the most central role in symptom maintenance. The findings of this study are also consistent with the existing literature on the importance of demographic characteristics, e.g. educational level and employment status, on antenatal mental health, and reflect known associations between lower physical health and environmental and social resources and higher distress levels (3, 8, 17–20, 25, 29–35). Centrality indicated that, among these factors, physical health emerged as a central component in the current model, underscoring the robust association between somatic and mental health. It also highlights the importance of a biopsychosocial approach to perinatal care (76).

In the current model, distress factors had a number of significant negative associations with different personal strengths, suggesting that directly targeting personal strengths may offer an efficient intervention strategy in promoting mental health in this population. More specifically, emotional balance demonstrated moderate-to-strong negative associations with two of three DASS components, and had relatively high strength centrality, indicating that it is a key direct protective factor in the network, especially against stress. Therefore, strengthening emotional balance could be a key focus in a positive psychological intervention for pregnant women. Self-confidence is also a promising target for intervention (40). It shows a direct negative association with depression, but moderate strength centrality and expected influence. This indicates that increasing self-confidence could have a specific effect on depressive symptoms. Resilience showed a somewhat different picture. It exhibited weak negative connections with all three DASS dimensions (32, 41, 43), indicating a weak direct protective effect. However, its high strength centrality and positive expected influence suggest it plays an important global protective role within the psychological network. These factors are strongly interconnected with physical health, contributing to the broader discourse on the impact of psychosocial health on pregnancy outcomes (11, 12).

Beyond personal strengths directly associated with lower distress, the network revealed a dense web of positive connections among other psychological strengths. Although many of these nodes had limited or no direct links to distress symptoms, their positive associations with more central protective factors suggest that they may indirectly support mental health. This highlights the value of targeting broader constellations of strengths in intervention, consistent with the resilience framework proposed by Wang et al. (41). Among these factors, identity is of particular significance due to its highly centralized and clustered nature. The findings of the present study indicate that identity exhibits a high degree of potential for cascading positive effects across the network, thereby leading to a reduction in distress. The results are consistent with a large body of theoretical models (77–79) and the alternative models of personality disorders of the current classification systems (49, 50) that emphasize the central role of a stable and coherent sense of self in healthy personality functioning.

Wisdom also exhibits strong centrality and appears to connect intrapersonal strengths with interpersonal factors, while also exerting a weak but negative direct effect on depression. Wisdom does not function primarily as a direct means of reducing distress; rather, it serves as a pivotal mediating resource that can mobilize other protective factors. Interventions that promote wisdom, such as finding meaning in life, developing insight, and perspective taking, can contribute to psychological well-being in an indirect yet effective manner. These findings are in line with the results of our previous study, in which wisdom was one of the most influential nodes, being highly connected to all higher-order factors and well-being (55). The present research contributes to the substantial body of existing literature on the association between wisdom and well-being, as well as positive emotions, and resilience across the lifespan (80).

These results align well with Self-Determination Theory (54), as identity and wisdom support the fulfilment of the basic psychological need for autonomy and the development of a coherent self. Self-confidence and emotional balance contribute to competence and emotional regulation, helping individuals navigate distress more effectively. Resilience reflects the capacity to sustain motivation and psychological well-being under stress, reinforcing autonomy, competence, and relatedness.

In the schema therapy model (46, 81), identity and wisdom are highly related to a strong Healthy Adult mode. Self-confidence and emotional balance serve as specific protective resources that counteract maladaptive schemas such as failure, emotional deprivation, or inhibition. Resilience may function as a global stabilizer, supporting recovery from schema activation and strengthening adaptive coping within the therapeutic process.

Interestingly, anxiety was positively associated with imagination and self-assertion, suggesting that some traits may function differently depending on their intensity or context. For example, the connection between imagination and anxiety may reflect the role of heightened mental imagery in anxious ideation (82). While these nodes had moderate centrality, their associations point to the complex interplay between cognitive and emotional traits in pregnancy.

Similarly, a weak yet significant positive association emerged between stress and empathy. It is plausible to assume that anxious individuals may be more sensitive to others’ emotions, which can enhance empathy. Anxious individuals might also tend to overly identify with others’ negative emotions, which can increase their anxiety. Furthermore, such individuals often exhibit heightened sensitivity to rejection and criticism (83). Although this connection was weak, it highlights how some protective traits may carry psychological costs under specific conditions.

### 4.2 Limitations

The study is subject to several limitations. The sample was imbalanced, with a disproportionate representation of women with high levels of education and living in relationships, which might have affected the results. However, the present sample was comparable to a large sample representative of pregnant women in Hungary in multiple domains, i.e., mean age, relationship status, parity and economic activity (3). Further research is needed to include samples with other characteristics. Although we recorded the date of the assessment and asked about the expected date of delivery, only women who agreed to participate in the follow-up assessment answered this question. However, in a subsample of 158 women, gestational age had little impact on the study variables. Therefore, this limitation is unlikely to have substantially influenced the results. We involved women in the second and third trimester of pregnancy; therefore, the results cannot be generalized for women in the first trimester. Participants were required to confirm that they were not undergoing neurological or psychiatric treatment at the time of the study. However, the implementation of structured interviews would have enhanced the precision of the study, as it would have facilitated the exclusion of participants with any current or lifetime psychiatric diagnosis. Although we did assess physical health in general, we did not assess pregnancy complications more specifically, which may independently elevate distress and skew results. Additionally, the proportion of unplanned pregnancies in the sample is unknown. The online recruitment method may have introduced selection bias, as it likely attracted pregnant women who are more health-conscious, technologically literate, or actively seeking information online. The study focused solely on expectant women; however, involving fathers and families may also be warranted (84). Cultural differences may also influence the findings, necessitating further research in diverse cultural contexts. Converging reviews indicate that social support may be a universal protective factor for perinatal mental health; however, its sources and expressions vary across cultural contexts (e.g., partner- vs. kin-based support, traditional postpartum practices, and the role of spirituality) (85). Resilience may also be considered a universal protective factor. Similar to our findings, a positive relationship with antenatal mental health has also been demonstrated in culturally different (e.g., in Spanish (86) and Chinese (87)) samples. Some other findings show a more complicated picture. Whereas the Polish postpartum study (88) identified assertiveness as a protective factor that negatively correlated with postpartum depression, our data showed a positive association between assertiveness (and imagination) and anxiety, suggesting that the role of assertiveness may vary by perinatal phase and cultural context. Conducting such research could assist in addressing the observed disparities in perinatal care across regions (84) and in fully implementing the World Health Organisation’s recommendations (76).

The use of self-report questionnaires may be subject to factors such as social desirability, insight, cognitive abilities, and mental health symptoms, potentially introducing bias. Both physical and mental health were assessed solely through self-reported measures. The lack of objective clinical data may affect the accuracy of the results, as participants’ subjective perceptions could introduce bias into the measurement of health status. Future research should employ alternative methods, such as behavioral observation, and utilize additional informants. The present study adopted a novel approach to examining personal strengths, a field that remains under-explored, with a paucity of research available on the BSS. Further studies are necessary to advance our understanding in this domain. For example, the convergent validity of the BSS should be assessed by examining its relationship to other well-known measures of strengths (89). The results could be affected by the decision to exclude the self-control subscale of the BSS from the analyses. As discussed earlier (55), this subscale captures diverse aspects of self-control — such as inhibition, delay of gratification, and distress tolerance — that demonstrate low inter-item correlations, suggesting they reflect distinct constructs rather than a unified factor; a narrower, goal-oriented definition could improve its reliability.

The study’s cross-sectional design did not allow for drawing causal conclusions. The relationships between mental health symptoms, quality of life, and social and personal factors may be bidirectional. Longitudinal studies are necessary to investigate their interrelations over time. Such studies could also test specific hypotheses on the mechanisms of specific interventions; for example, it would be worth exploring the mediators through which identity and wisdom may indirectly affect symptoms of distress. It would be beneficial to expand the study’s design to encompass psychophysiological markers. The psychological protective factors identified by our research are plausibly embedded in well-described biological mechanisms. Emotion regulation and resilience have been demonstrated to modulate HPA-axis reactivity, resulting in more adaptive cortisol dynamics (90). Perinatal dysregulation of this axis has been associated with depressive and anxious symptomatology. Furthermore, the oxytocinergic system, a critical substrate of maternal bonding and stress alleviation, has been linked to perinatal mood outcomes. However, findings remain heterogeneous (91). Finally, alterations in top-down fronto-limbic circuitry may contribute to both risk and protection; for example, more adaptive cognitive reappraisal shows measurable neural correlates in the postpartum period (92). Further longitudinal, multimethod research is necessary to assess these pathways and elucidate the causal mechanisms underlying them.

## 5 Conclusion

The strong interconnections between various protective factors and mental health suggest the necessity of a holistic approach in prenatal care. The findings of this study demonstrate that multifaceted interventions targeting social, physical, environmental, and personal strengths can enhance maternal mental health during pregnancy.

The results of this study indicate that, regarding personal strengths, the primary objectives of an intervention should encompass the enhancement of emotional regulation and mood stability, the strengthening of resilience to effectively manage stress, fear and uncertainty and the augmentation of confidence in navigating pregnancy, childbirth, and motherhood. Through structured daily activities including mindfulness, cognitive reframing, and strength-based activities, participants build practical tools for managing stress and navigating the journey of motherhood. Furthermore, such an intervention may also concentrate on more distal protective factors, such as identity and wisdom. Identity in pregnant women can be fortified through values-based reflection exercises and guided journaling focused on the evolving role of motherhood. This approach is hypothesized to help them build a coherent and resilient sense of self. The cultivation of wisdom can be facilitated by group discussions centered on past life challenges and decision-making processes. These discussions encourage the processes of meaning-making and perspective-taking, which may, in turn, contribute to a reduction in distress symptoms. In addition to existing positive psychological interventions (45), the Healthy Adult Strengths Tool of the iModes System (47) may serve as a useful framework for such an intervention.

## Data Availability

The datasets presented in this study can be found in online repositories. The names of the repository/repositories and accession number(s) can be found below: The datasets analyzed for this study have been uploaded and are freely available in the OSF: https://osf.io/mzxgv/?view_only=f4a2218ac42d41a983684046aaf3fe76.
